# Exploring plant-microbe interactions in adapting to abiotic stress under climate change: a review

**DOI:** 10.3389/fpls.2024.1482739

**Published:** 2024-11-15

**Authors:** Ali Muhammad, Xiangjun Kong, Shuaichao Zheng, Na Bai, Lijie Li, Muhammad Hafeez Ullah Khan, Sajid Fiaz, Zhiyong Zhang

**Affiliations:** ^1^ Henan Collaborative Innovation Center of Modern Biological Breeding, Henan Institute of Science and Technology, Xinxiang, China; ^2^ State Key Laboratory of Crop Stress Adaptation and Improvement, College of Agriculture, Henan University, Kaifeng, China; ^3^ Institute of Molecular Biology and Biotechnology, The University of Lahore, Lahore, Pakistan

**Keywords:** climate factors, rhizosphere, nutrient acquisition, phytohormones, sustainable ecosystem

## Abstract

Climatic change and extreme weather events have become a major threat to global agricultural productivity. Plants coexist with microorganisms, which play a significant role in influencing their growth and functional traits. The rhizosphere serves as an ecological niche encompassing plant roots and is a chemically complex environment that supports the growth and development of diverse plant-interactive microbes. Although plant-microbe interactions have been extensively investigated however, limited exploration have been made how abiotic stresses affect the structure and assembly of microbial communities in the rhizosphere. This review highlights climate change influence on plant growth, functional traits, and microbial communities. It explores plant mechanisms for mitigating abiotic stress, such as removing reactive oxygen species (ROS), regulating antioxidant activity and indole-3-acetic acid (IAA) production, and controlling growth-inhibitory ethylene levels through colonization by bacteria producing ACC deaminase. Additionally, we elaborated the systematic communicatory network steered by hormonal crosstalk and root exudation, which can modulate and initiate the dialogues between plants and surrounding microbes. This network ultimately promotes the chemotactic movement of microbes towards the rhizosphere, facilitating their early colonization. Finally, we reviewed the recent advancements for understanding how plant-microbe interactions foster resilience under climate stress.

## 1 Introduction

Climatic trends show a significant increase in global mean temperature since 1970s, coupled with shift in precipitation patterns, and the frequency of extreme weather events (Capua and Rahmstorf, 2023). Agriculture sector is directly affected by climatic factors, experiencing a significant reduction in farm productivity. In addition, climate change could expand the range of pathogens and pests, leading to more frequent and severe disease outbreaks (De Wolf and Isard, 2007; Garrett et al., 2021). Recent findings has shown that global agricultural production is highly vulnerable to abiotic stresses (Bowerman et al., 2023). Furthermore, to meet the demand of growing human population, about 50% increase in agricultural production is needed by 2029 (Nawaz et al., 2024b). Consequently, a significant increase in deforestation and loss of natural habitats is occurring to acquire more land for cultivation (Foucher et al., 2024). To ensure food security with a limited expansion of agricultural land, a sustainable strategy involves improving the resilience of plants to climate change. Therefore, plant growth-promoting microorganisms represents one of the valuable resources to explore for increasing farm productivity (Bender et al., 2016).

Plants are associated with a diverse group of microorganisms, responsible for many essential functions e.g., plant growth, root development, nutrient use efficiency, and promoting resistance to abiotic and biotic stresses (Ojuederie et al., 2019). The rhizosphere serves as the central hub for the interactions among plant roots, soil, microorganisms, and environment (Trivedi et al., 2020). It is a specialized layer of soil around the root system, and the microbes in the soil are referred to as rhizosphere microorganisms (Rout and Southworth, 2013). Macro-nutrients such as nitrogen, phosphorous, and potassium, are assimilated by plants through the rhizosphere, enabling their integration into the nutrient cycle (Thepbandit and Athinuwat, 2024). Plants establish beneficial associations with various microorganisms in the rhizosphere to shape their community composition through rhizosphere deposition (Toju et al., 2018). Several important symbiotic microorganisms have been identified to promote plant growth by reducing the occurrence of different plant diseases, the regulation of phytohormones, and enhancing nutrient acquisition (Kuypers et al., 2018; Hu et al., 2020; Lopes et al., 2021). Plant growth-promoting rhizobacteria (PGPR) act as biofertilizers by enhancing the availability of both macro and micronutrients, thereby improving crop yield and soil fertility (Lopes et al., 2021). Therefore, a critical understanding of plant-microbe interactions in the rhizosphere, steered by hormonal crosstalk and root exudation, and their combined application for improving crop productivity is required.

Abiotic stresses impact not only plant physiology and metabolism but also soil microorganism activities. However, the impact of stress varies depending on the time, host plants, intensity, and other environmental factors (Georgieva and Vassileva, 2023). For instance, a significant reduction in growth and grain yield was observed in wheat grown under drought conditions, primarily due to the negative impact on photosynthesis, leaf area, seed set and weight (Rahimi-Moghaddam et al., 2023). In contrast, *Thymus serphyllum* exhibits increased production of osmolytes, such as proline, sorbitol, mannitol, and other amino acids, which confer tolerance to drought stress (Moradi et al., 2017). Furthermore, under salt stress, the rhizosphere of groundnuts exhibited a notable presence of Acidobacteria and Cyanobacteria, enhancing their salt tolerance (Xu et al., 2020). On the other hand, rice plants revealed significant yield penalties, including reduced growth, germination, and tillering which in turn affected plant biomass and height (Fang et al., 2023). Thus, interactions between plants and microbes during abiotic stress conditions are dynamic and complex. In order to harness the potential of plant microbiota in agriculture, it is essential to understand the impact of abiotic stressors on plants, microorganisms, and plant-microbe interactions (Fadiji et al., 2023). In the present review, we have summarized the effects of changing climatic conditions on plant-microbe interactions and highlighted the positive roles of plant associated microbes in enhancing agricultural production. Additionally, we discuss recent advancements in mitigating different stressors and their significance for achieving key objectives in future research.

## 2 Deciphering the coexisting relationship between plants and soil microbiota

Plants are associated with a varied and taxonomically organized community of microbiomes i.e., viruses, fungi, bacteria, and archaea that co-exist with plants in the rhizosphere (root-surrounding soil), endosphere (internal tissue), and phyllosphere (above-ground parts) to perform important activities regulating host health and fitness (Afridi et al., 2022). Among these micro-environments, rhizosphere is one of the most complex and diverse habitats for microbial communities. Plant-associated microorganisms may come from different sources, including soil, seeds, water, and other neighboring organisms i.e., insects and animals. Some of these organisms can develop a complex symbiotic relationship with plants (Fitzpatrick et al., 2018). Specifically, plant-associated microbiome form a symbiotic unit known as “holobiont” which can be influenced by environmental factors (Trivedi et al., 2020). A holobiont is a complex and interconnected system of organisms, living together in a close association in all types of ecosystems (Matthews, 2024). These microorganisms within the holobiont can significantly improve plant health by enhancing mineral solubility (Lemanceau et al., 2017), altering the signaling of phytohormones such as auxin (IAA), gibberellin (GA), and cytokinin (CK) (Spaepen and Vanderleyden, 2011), and directly providing nutrients (Saleem et al., 2024), alongside strengthening resistance against phytopathogens (Jin et al., 2024).

**Figure 1:**
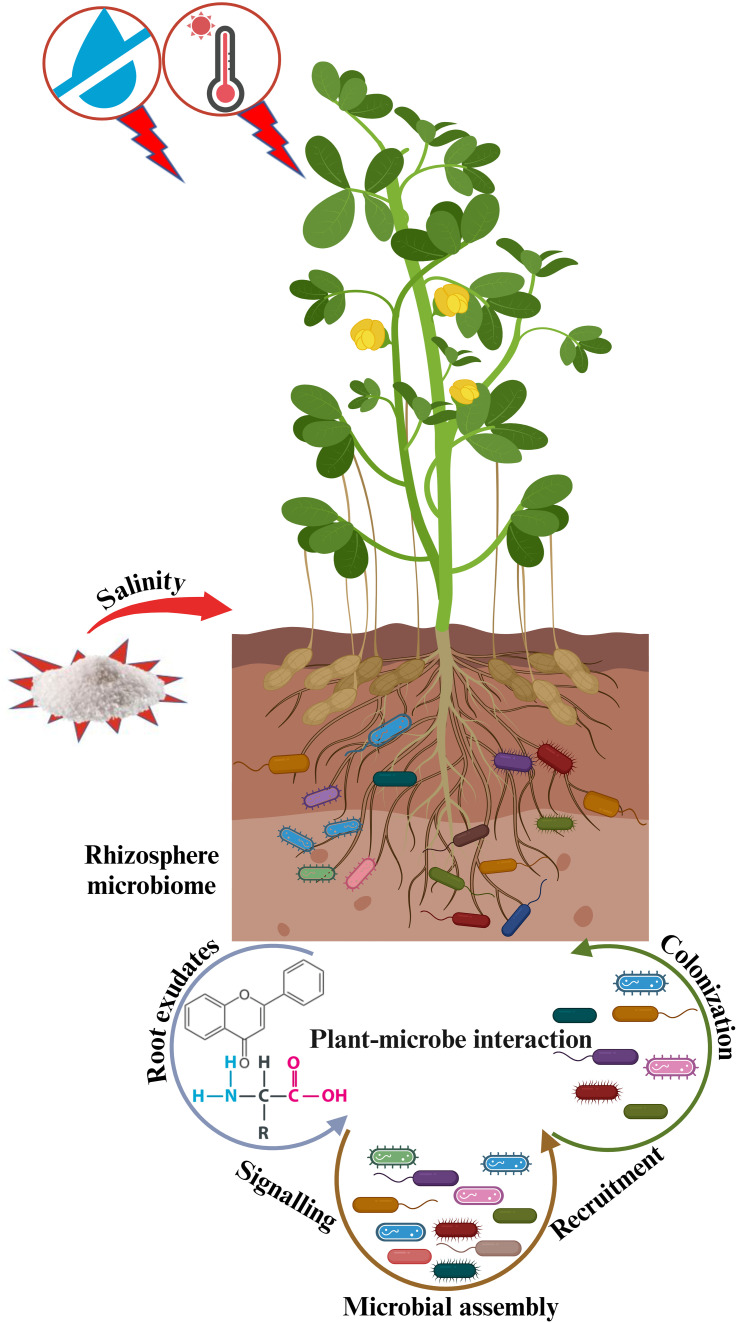
Systematic overview of plant-microbe interaction to mitigate abiotic stresses in changing climates. Plants release root exudates, such as flavonoids and amino acids that attract nearby microbes to their roots, helping the plants optimize water and nutrient allocation for better growth and survival in unfavorable circumstances.

Plant-microbe crosstalk initiates with the release of chemical signals i.e., flavonoids and amino acids, establishing a favorable environment where microbes ultimately reside and assist plants in coping with stress and regulating growth (Stefan et al., 2018) (**Figure 1**). Therefore, plants recognize signals created by beneficial microbes during the early phases of symbiosis (Ravelo-Ortega et al., 2023). According to Fadiji et al. (2023), when plants receive adequate water and nutrient supply, rhizosphere microorganisms consistently aid in helping plants adapt to abiotic stresses, thereby potentially enhancing crop yield. At present, there are several studies on the isolation, identification, and application of useful symbiotic microorganisms as an alternative to chemical fertilizers, that are hazardous to humans, animals, aquatic lives, and environment (Kumar et al., 2022; Shahwar et al., 2023). Abdelaziz et al. (2019) demonstrated significant improvements in tomato growth and yield by introducing the root endophytic fungus (*Piriformospora indica*) into the soil. The interactions between plants and rhizobia are very specific and exist at a level of species and genotype, leading to the formation of effective symbiotic relationships (Wang et al., 2018). Overall, the rhizobia are the key members in the soil to produce and fix nitrogen compounds that help plants to grow and survive under unfavorable circumstances for better ecosystem productivity.

## 3 Climate-associated abiotic stresses alter plants and their associated microbes in the rhizosphere

Plants are consistently exposed to the external environment that constantly changes in many ways (Nawaz et al., 2024a). Certain regions of the world are already facing extreme climatic shifts, such as drought, salinity, and extreme temperatures. These climatic stresses have detrimental effects on plant-associated microbial communities, which in turn influence plant growth and development (Afridi et al., 2022). In response to unfavorable conditions, plants induce a wide range of physiological and morphological modifications to adapt to these abrupt changes thus, experiencing significant growth and yield penalties (Xu et al., 2023). Abiotic stresses can affect rhizosphere microbial communities in various ways including the alteration in composition and function of microbial population in the rhizosphere. For instance, prolonged exposure to drought stress can lead to an increase in the abundance of Actinobacteria and Firmicutes (Vescio et al., 2021). Additionally, a proportionate rise in the abundance of Acidobacteria and Cyanobacteria was observed in the peanut rhizosphere, when exposed to salt stress (Xu et al., 2020). Recently, Dollete et al. (2024) revealed a reduction in symbiotic nitrogen fixation in forage legumes, thereby effecting plant growth and development. Furthermore, altered phosphate solubilizing efficiency of the biocontrol fungus *Trichoderma* sp. was observed under abiotic stress conditions such as pH, temperature, and heavy metals (Rawat and Tewari, 2011).

Plant growth promoting microorganisms (PGPMs) have been frequently investigated to mitigate certain abiotic stresses while enhancing plants adaptation under unfavorable conditions (Chieb and Gachomo, 2023; Kibret et al., 2024). To sustain their growth under stressed conditions, plants employ various strategies i.e., induction of chemical signaling and regulation of phytohormones (biochemical adaptation), stomatal closure (physiological adaptation), changes in growth pattern (morphological adaptation) (Naamala and Smith, 2020) (**Table 1**). Climate-associated abiotic stresses may influence plants in different ways and at different growth stages, limiting plant performance (Lata et al., 2018). These abiotic stresses are discussed in detail to further elaborate their detrimental effects on plant adaptation, which may hinder the development of a sustainable ecosystem and reduce agricultural production.

**Table 1:** Plant growth promoting microorganisms enhancing abiotic stress tolerance through various mechanisms.

Plant specie	PGPMs	Plant-microbe interaction mechanism	Improved plant traits	References
Temperature stress				
Soybean	*Bacillus cereus*	Enhanced production of phytohormones (GA, IAA) and organic acids	Biomass, and chlorophyll content	(Khan et al., 2020a)
Soybean	*Paecilomyces formosus* LHL10, *Penicillium funiculosum* LHL06	Improved nutrient uptake, upregulation of antioxidant enzymes to reduce lipid peroxidation	Plant growth attributes and photosynthetic activity	(Bilal et al., 2020)
Tomato	*Paraburkholderia phytofirmans*	Enhanced accumulation of sugars, total amino acids, proline, and malate	Chlorophyll content, and gaseous exchange	(Issa et al., 2018)
Wheat	Enterobacter sp. SA187	Modifying the trimethylation of lysine 4 on histone H3 (H3K4me3)	Agronomic traits, biomass, and grain yield	(Shekhawat et al., 2021)
Rice	*Brevibacterium linens* RS16	Regulation of antioxidant enzymes, heat shock proteins, and ethylene emission	Plant growth, and thermotolerance	(Choi et al., 2022)
Rice	*Rhizobium* sp. IIRR N1, *Gluconacetobacter diazotrophicus*	Enhanced antioxidant enzymes (viz. SOD, CAT, APX)	Chlorophyll content, root and shoot biomass	(Chaganti et al., 2023)
Drought				
Peanut	*Bradyrhizobium* sp. SEMIA6144	Increased soluble sugar and ABA contents	Plant growth and chlorophyll content	(Furlan et al., 2012)
Maize, Peanut	*Enterobacter* sp. J49	Improved nitrogen fixation and IAA production	Pod and grain yield	(Anzuay et al., 2023)
Pea	*Bacillus thuringiensis* MH161336	Regulation of antioxidant enzymes to decrease lipid peroxidation and ROS	Plant height, seed weight, number of leaves, and pods	(Arafa et al., 2021)
Broccoli	*Variovorax* sp. YNA59	Increased SA level and antioxidant enzymes activities	Plant growth attributes, chlorophyll content, and moisture content	(Kim et al., 2020)
Wheat	*Burkholderia phytofirmans* PsJN	Improved ionic balance and antioxidant levels	Grain yield, photosynthetic rate, water use efficiency and chlorophyll content	(Naveed et al., 2014)
Salinity				
Rice	*Bacillus pumilus* JPVS11	Increased IAA, ACC deaminase activity and EPS production	Plant growth, and chlorophyll content,	(Kumar et al., 2021)
Rice	*Halobacillus dabanensis* strains SB-26, GSP 34	Nitrogen fixation and IAA production	Root length, shoot height, total weight, and chlorophyll content	(Rima et al., 2018)
Maize	*Bacillus* sp. PM31	Improved radical scavenging capacity, antioxidants and upregulation of stress-related genes (APX and SOD)	Agro-morphological traits	(Ali et al., 2023)
Pea	*Bacillus subtilis* RhSt-71*, Bacillus safensis* RhStr-223, and *Bacillus cereus* RhStr-JH5	Increased antioxidant enzymes, IAA synthesis, Phosphate solubilization, siderophore, and ammonia production	Chlorophyll content and plant growth	(Gupta et al., 2021)
Wheat	*Bacillus megaterium* strain PN89	Higher Phosphate solubilization, and IAA, siderophore, and protease production	Germination percentage, root and shoot length, and other growth attributes	(Lee et al., 2021)

GA, gibberellin; IAA, indole-3-acetic acid; SOD, superoxide dismutase; CAT, catalase; APX, ascorbate peroxidase; ABA, abscisic acid; ROS, reactive oxygen species; SA, salicylic acid; ACC, 1-aminocyclopropane-1-carboxylate; EPS, extracellular polymeric substance.

### 3.1 Harnessing plant-microbes interplay to alleviate temperature stress

Temperature serves as a fundamental factor influencing plant development and phenological features together with shaping the microbial community associated with the plants (Kashyap et al., 2017). Compant et al. (2010) suggested a projected increase of about 1.8-3.6°C in global mean temperature by the year 2100 resulting water scarcity across several regions of the world which may significantly influence the composition, activities, and distribution of rhizosphere microbiome (Farooq et al., 2022). A substantial shift in microbial respiration rate in response to higher temperatures may accelerate their growth and abundance (Classen et al., 2015). Similarly, Vargas (2024) revealed that increased temperature may lead to an exponential increase in soil respiration. Furthermore, higher temperatures may influence the utilization of organic matter by microorganisms (Frey et al., 2013). Moreover, Velásquez et al. (2018) reported the connection between the pathogenicity of microorganisms with temperature changes. Higher temperatures may also mediate bacterial virulence, such as *Pectobacterium atrosepticum* causing the occurrences of soft rot disease and further affects the degradation of cell walls, resulting in an increased disease incidence in plants (Hasegawa et al., 2005).

Temperature stress greatly affects microbial activities as they require an optimal range of temperature for sustaining their growth, reproduction, and ability to cause diseases, unless they quickly acclimatize to cope with temperature changes (Wang et al., 2021a). For example, several microbes have developed specialized adaptations to counter extreme environmental conditions while supporting their host plants by efficiently utilizing carbon allocation (Vera-Gargallo et al., 2023). They also contain specific enzymes and complex regulatory networks of secondary metabolites to overcome the adverse effects of temperature stress (Raddadi et al., 2015). Several thermotolerant microbes i.e., endophytic bacteria and arbuscular mycorrhizal fungi (AMF), modify their structures to survive under high temperature and protect their host plants from extreme environmental conditions. Rasmussen et al. (2020), studied the impact of temperature on plant-associated AMF colonization and plant performance and found that under continuous temperature increase, both fungal colonization and plant growth increase. Several experimental studies reported similar findings about the increased colonization of AMF in response to rising temperatures (Compant et al., 2010; Xie et al., 2024). Khan et al. (2020a) revealed the positive effect of plant growth-promoting endophytic bacteria on crop production under higher temperatures and suggested their use as biofertilizers to mitigate heat stress damage in soybean plants. Additionally, other studies reported the use of thermotolerant bacteria enhancing the content of chlorophyll in rice and canola (Glick et al., 1997; Chaganti et al., 2023).

Moreover, certain microbes may help plants to cope with multiple stresses i.e., *Burkholderia phytofirmans* PsJN strain has been reported to enhance heat tolerance in tomatoes, salinity and freezing resistance in *Arabidopsis*, cold resilience in grapevines, and drought tolerance in wheat (Miotto-Vilanova et al., 2016; Issa et al., 2018). Similar findings have also been reported for other crops, indicating that heat-resistant bacteria could enhance crop growth and development in wheat, rice, tomato, potato, chickpea, sorghum, and canola plants under heat stress (**Table 1**). Altogether, it is crucial to understand the effects of elevated temperature on plant-microbe interactions, and to identify target microbes and their association with crops under a specific environment for sustainable ecosystem with improved agricultural production.

### 3.2 Drought and the role of plant-microbe symbiosis

Drought is one of the major causes of agricultural losses worldwide, threatening food security. Climate change and global warming will further intensify the situation with severe drought episodes, posing a greater threat to agricultural sustainability (Fadiji et al., 2023). One of the noticeable impacts of climate change is higher variation in precipitation patterns, which directly influences the moisture content in the atmosphere and soil, resulting in flood or drought conditions (Tabari, 2020). Water scarcity leads to several physiological and morphological responses in plants as a consequence of drought (Kumar and Verma, 2018). Drought limits the normal growth and development, disturbs water requirements, and reduces water use efficiency in plants. During the seedling stage, drought stress in plants maintains root development while limiting shoot growth, leading to a higher root/shoot ratio (Kou et al., 2022). Additionally, extreme water-limiting conditions cause retarded leaf development due to the shrinkage of the plant cells which ultimately reduces turgor pressure, resulting a decline in plant fresh weight (Fahad et al., 2017). The root morphology also adapts certain modifications such as shrinking, to optimize the distribution of water and nutrients to several parts of the plant under drought conditions, thus preventing the loss of water that might affect leaves potential to utilize photosynthesis II (Ma et al., 2020). Prolonged water scarcity affects cell wall integrity, resulting in the generation of ROS, causing premature leaf senescence, promoting ethylene accumulation, affecting chlorophyll content, and suppressing photosynthetic mechanisms (Kumar and Verma, 2018). In addition, drought impairs the dissolution and translocation of salts, leading to their accumulation in the rhizosphere soil, which ultimately causes salinity stress (Gupta et al., 2022).

**Figure 2:**
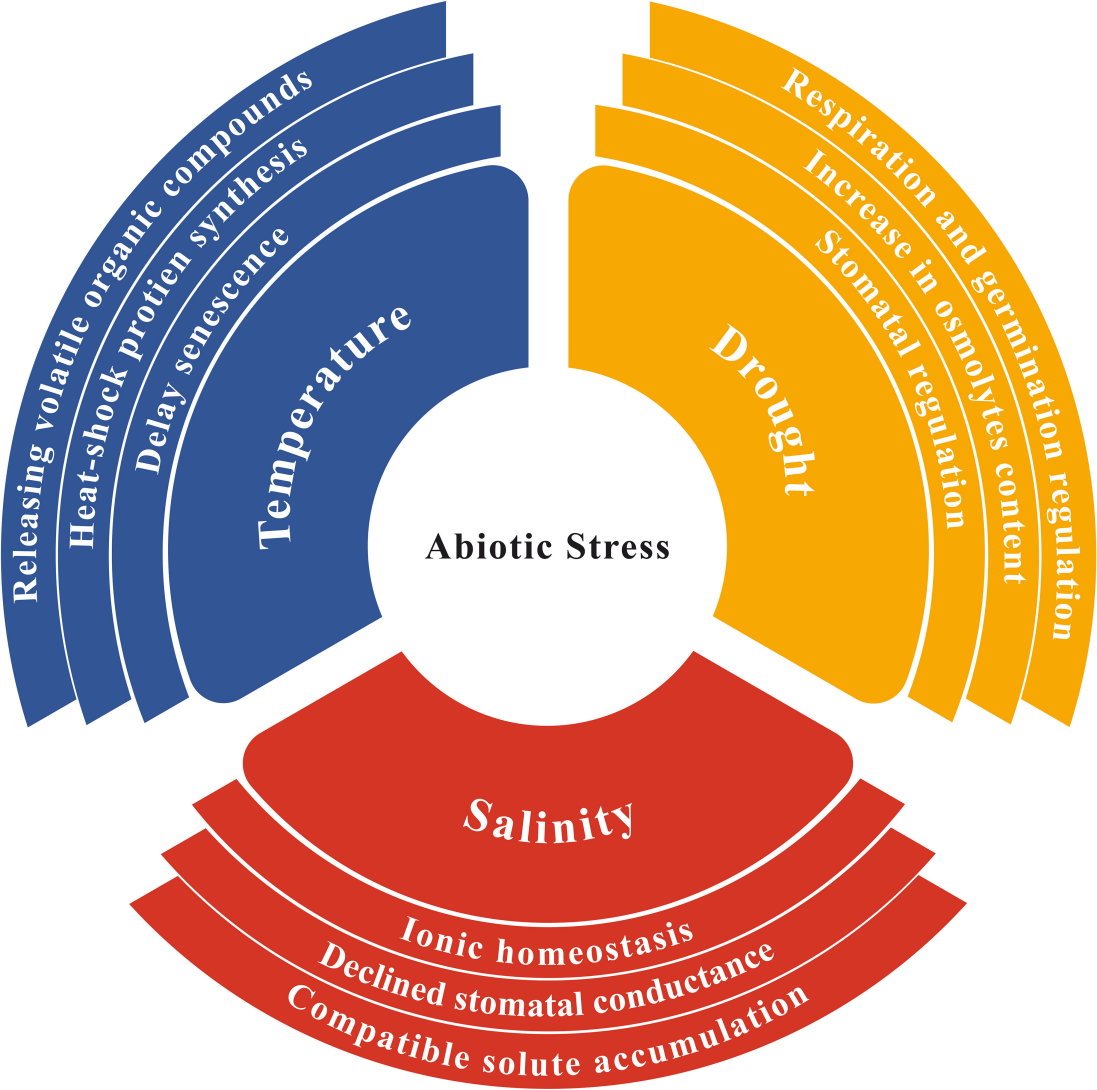
Plant adaptation and utilization of certain protective mechanisms against abiotic stresses in changing climates.

Several plant species faced drought conditions for a long time have modified several traits to tolerate drought conditions, e.g. better utilization of phytohormones, increased synthesis of osmolytes, and heat-shock proteins to sustain growth and yield (**Figure 2**). These plants also activate cellular mechanisms to maintain water and salt balance by transporting excessive salt from specific cells to other parts of plant, producing suitable solutes that promote drought tolerance and increase several compatible antioxidant enzymes for scavenging excessive ROS (Gupta et al., 2022). During a metabolomic analysis, it was revealed that *Thymus serphyllum* exhibits increased production of osmolytes, such as proline, sorbitol, mannitol, and other amino acids conferring drought stress tolerance (Moradi et al., 2017). Drought significantly influences soil characteristics and the microbial community in the rhizosphere (Manzanera, 2021). The root system and rhizosphere microorganisms influence each other through mutual interactions (**Figure 1**).

Rhizosphere microorganisms through symbiotic interaction greatly reduce the effects of drought by improving root architecture and facilitating water and nutrient absorption. This association also promotes the release of certain beneficial metabolites that may help plants in enhancing the root/shoot ratio, promoting yield with greater biomass production, improving water absorption capacity, and enhancing nutrient availability (**Figure 1**) (He et al., 2021; Rawal et al., 2022). The host plant in turn, initiates root exudation, which generates other factors that affect the performance of these microorganisms (Gupta et al., 2022). Consequently, manipulating rhizosphere microbiomes can boost the capacity of crops in stress reduction (Xu and Coleman-Derr, 2019; de Vries et al., 2020). For instance, certain plant species can tolerate higher drought by allocating more endosphere *Streptomyces* (Fitzpatrick et al., 2018). Similarly, sorghum seedlings with *Streptomyces* colonization resulted in increased root growth under drought, while no noticeable effects were observed in well-watered conditions (Xu et al., 2018). Furthermore, AMF significantly contributes to the water availability of crops by utilizing external hyphae and glomalin, thereby enhancing crop drought tolerance (Wang et al., 2023). Likewise, AMF has a prominent role in root growth and development in citrus, and maize under arid soil conditions (Wu et al., 2013; Zhao et al., 2015). Several similar studies reported the role of PGPMs in plant defense against several abiotic stresses (**Table 1**). These findings revealed that plant health is greatly dependent on the composition and activity of plant-associated microbiome, thereby harnessing rhizosphere microorganisms under drought stress may have significant effects on crop yield by optimizing water and nutrient allocation.

### 3.3 Interactive mode of plant–microbiota in mediating salt tolerance

Soil salinity is one of the major abiotic stress, exerting adverse effects on crop growth and yield (Julkowska and Testerink, 2015). It has been estimated that approximately 7% of the total global fields (1 billion hectares of soil) are affected by salinization (Bayabil et al., 2021). More seriously, the area occupied by salinity-affected soils is increasing by around 10% per year due to climate change, owing to less precipitation and increased surface evaporation, improper agricultural practices (increased use of fertilizers and saline water irrigation, and industrial pollution) (Ouhibi et al., 2014; McFarlane et al., 2016). Naturally, plants are adapted to absorb a reasonable quantity of soluble salts resulting from mineral weathering. However, inadequate precipitation hinders salt leaching, causing the deposition of soluble salts in the root zone (Shrivastava and Kumar, 2015). Additionally, the intrusion of saltwater through surface or groundwater connections may have escalated the problem of salinity (Bayabil et al., 2021). Furthermore, the higher salt concentration in the rhizosphere influences water and nutrient absorption, leading to osmotic stress. This condition causes oxidative damage by inducing an increased number of ROS (Isayenkov and Maathuis, 2019; Rahman et al., 2024). Additionally, soil salinity has adverse effects on the nodulation process, reducing crop production and hindering nitrogen fixation by decreasing nitrogenase activity (Kumar and Verma, 2018).

When facing saline conditions, plants may develop several defensive strategies to safeguard themselves e.g., developing salt-releasing glands or trichomes (Yuan et al., 2016), restoring ionic homeostasis, osmotic and ROS levels (Yang and Guo, 2018), adjusting stomatal conductance (Li et al., 2020) and regulating certain growth patterns like flowering time (Kazan and Lyons, 2016) (**Figure 2**). Soil microorganisms greatly contribute to plant tolerance against salt and drought stresses through certain plant-mediated mechanisms (Sangiorgio et al., 2020) (**Table 1**). Microorganisms also accelerate antioxidant responses to protect plants from oxidative injury. These adaptive responses are prevalent in bacteria as they enable them to survive numerous harsh conditions (Liu et al., 2019). Among these features, extracellular polymeric substance (EPS) production regulates several services including mass transfer restriction, preventing water loss, and regulating essential biomolecules such as enzymes, nucleic acids and exopolysaccharides (López-Ortega et al., 2021). EPS-producing microbes can enhance salt tolerance in plants by absorbing sodium ions in the soil, making them less available to plants (Bhagat et al., 2021). Additionally, bacterial exopolysaccharides play a prominent role in improving soil structure under salt stress by producing micro and macro-aggregates (Grover et al., 2011).

Halophiles or halo-tolerant represent a diverse group of microorganisms with a remarkable ability to grow in a wide range of NaCl concentrations (Abbas et al., 2019). These microbes can be categorized ranging from highly halophilic (2.5-5.2 M) to halotolerant (0.3-0.5 M) through various adaptive mechanisms (López-Ortega et al., 2021). Some of the major contributions of these microbes include ACC deaminase activity, EPS production, nitrogen fixation, IAA production, biofilm development, building-up osmolytes in the cytoplasm of plant cells, maintaining turgor pressure in salt-stressed cells, and limiting osmotic and oxidative stress by producing plant hormones and antioxidants (**Table 1**) (Rajput et al., 2018; Rima et al., 2018; Nawaz et al., 2020; Kumar et al., 2021). According to Li et al. (2021), a diverse microbial community was recruited by plant roots in the rhizosphere of the salt-treated plants, enhancing plant salt tolerance. Similarly, the rhizosphere of groundnuts exhibited a notable presence of Acidobacteria and Cyanobacteria, when exposed to salt stress (Xu et al., 2020). Even though these findings reveal the importance of microbial communities in the defense mechanisms of plants under salt stress, further in-depth mechanistic investigation using different plant species under saline condition are required to fully understand the effects of salt stress on the plant-associated microorganisms.

## 4 Signaling and cross-talk involved in plant-microbe interactions

Plants in their natural habitat engage in dynamic crosstalk with various environmental signals, facilitating the integration of microbial communities. In such communicatory networks, plants possess the ability to efficiently sense and respond to interactive stimuli. However, upon recognizing of microbial substances, they have the potential to establish symbiosis or develop immune responses. Overlooking this complicated web of communication networks, the significance of chemical signaling in perception and modulation is highly pivotal for stationary organisms like plants (Afridi et al., 2022). Plants utilize chemical signals as stimuli to establish beneficial relationships with surrounding microorganisms, either aboveground (trunk, shoots, leaves) or belowground (roots). This sophisticated communicatory network is steered by hormonal crosstalk and root exudation, regulating the intricate interactions between plants and their diverse biotic and abiotic environments.

### 4.1 Role of root exudates in shaping rhizosphere microbiota

During various growth stages, plants exudate a wide array of metabolites including primary and secondary metabolites, through their roots. Primary metabolites regulate processes including growth, development, and nutrient acquisition. On the other hand, secondary metabolites are generally not involved in plant survival; however, they serve essential functions in plant defense and protection against insects and pests. Additionally, secondary metabolites are important for plant adaption to changing environments and resilience to different biotic and abiotic stresses. A diverse range of defensive functions are performed by these secondary metabolites under both biotic and abiotic stresses including photoprotection, signaling, antimicrobial ability and structural stabilization (Ingle et al., 2016). Moreover, plant secondary metabolites contribute to pest and disease tolerance, act as signaling molecules for plant-microbe interactions, and influence microbial communities associated with their hosts (Fakhri et al., 2022).

The chemical composition of root exudates is greatly dependent on the genetics and age of the plant species (Zhang et al., 2021) as well as the soil physico-chemical properties (Sasse et al., 2020). Variation in the root exudates among different plant species is mainly dependent on the intricate regulation of root system architecture (RSA) (Galindo-Castañeda et al., 2024). The area below the root cap is recognized as the major exudation site, although various root zones have been reported active in different plant species (Haldar and Sengupta, 2015). Furthermore, different root parts have different exudations. For instance, the root meristem and elongation site are responsible for asparagine and threonine, the root hair zone exudes glutamic acid, valine, leucine, and phenylalanine, whereas the whole root secretes aspartic acid (Haldar and Sengupta, 2015). Plant root exudation is the central source of nutrients in the rhizosphere, attracting indigenous microbes that thrive in that specific environment (Pascale et al., 2020). Root exudates not only function as signaling molecules, such as chemoattractants and stimulants, but in certain situations, they act as repellents and inhibitors. These chemicals continuously evolve with the changes in their immediate environment, modulating the initial dialogue between plant roots and soil microorganisms (Wiesenbauer et al., 2024). Chemotaxis and colonization are necessary for the establishment of a microbiome in the rhizosphere. Root exudates are considered the central source of signaling molecules for microorganisms, promoting their chemotactic movement toward the rhizosphere and facilitating early colonization. Thus, root exudates play key role in plant-soil feedback by modifying microbial communities.

### 4.2 Hormonal crosstalk in plant-microbe symbiosis for enhanced stress tolerance

**Figure 3:**
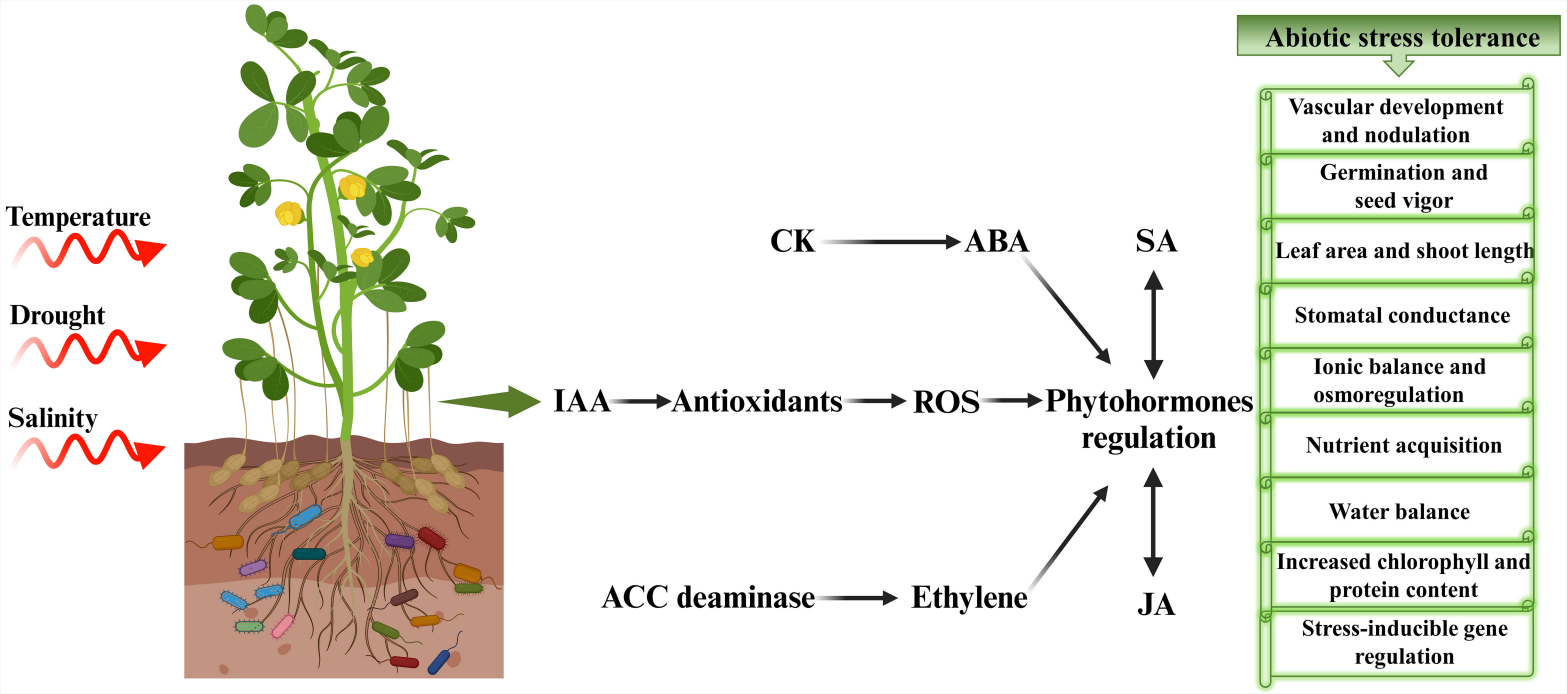
Hormonal crosstalk and signaling mediating abiotic stress tolerance in plants. IAA promotes antioxidant activity by neutralizing ROS. CK works with ABA to modulate stress responses. ABA is crucial for regulating stomatal conductance and promoting drought tolerance. SA interacts with other hormones like ABA and JA, influencing plant defense mechanisms. ACC deaminase modulates ethylene levels to mitigates stress effects. IAA, Indole acetic acid; ROS, reactive oxygen species; CK, cytokinins; ABA, abscisic acid; ACC, 1-aminocyclopropane-1-carboxylate; SA, salicylic acid; JA, jasmonic acid.

Plants growing under natural conditions constantly interact with both biotic and abiotic environments. To ensure their survival and to sustain the effects of these diverse and often hostile conditions, plants developed a sophisticated and adaptable environmental signaling network steered by phytohormones (Jain et al., 2021). This elaborate hormonal crosstalk fine-tunes plant responses to extremely dynamic and varied circumstances. Plant hormones are organic substances produced in small amounts that serve as important regulators of plant developmental processes in response to external stimuli by triggering different physiological mechanisms (Hirayama and Mochida, 2022). Plants naturally synthesize a multitude of hormones including auxins, GA, CK, and ABA to oversee their growth and metabolism (Iqbal et al., 2022). Studies indicate that phytohormone application during stress greatly enhances plant functioning and metabolic processes. Among phytohormones, auxin and ABA have pivotal roles in alleviating abiotic stresses (Hu et al., 2013). However, researchers have also deciphered the potential roles of other phytohormones in numerous studies (Singh and Singh, 2017; Sharma et al., 2019). The crosstalk of ABA, SA, jasmonates, and ethylene with the major growth-2promoting hormones such as auxins, GA, and CK, greatly contributes to plant defense against several abiotic stresses (**Figure 3**).

Microorganisms establish symbiotic relation with plants to protect them against abiotic and biotic stresses by triggering phytohormone signaling and activating defense mechanisms (Khan et al., 2020b). However, it is noteworthy that the influence of microbes on the regulation of phytohormones has significance not only in directly promoting plant growth but also in several other aspects of microbial effects on plants, such as enhancing nutrient acquisition and mediating stress resistance. Phytohormones play a significant role in mitigating abiotic stress by regulating vital functions in plants, including nutrient acquisition, water balance homeostasis, tolerance against pathogens, antioxidant activities, increased chlorophyll and protein content and stress-inducible gene regulation (**Figure 3**).

It is worth mentioning that microbes can modulate mineral acquisition through nitrogen fixation or phosphate solubilization, thereby indirectly influencing the concentration of phytohormones in plants, as the status of phytohormones partially contingent on the efficiency of nutrient acquisition (Kudoyarova et al., 2015). Moreover, the role of rhizosphere microorganisms in modulating plant hormonal status is likely involved in the majority of well-established mechanisms through which microbes promote plant growth. This could include activities like engineering the interplay of phytohormones or integrating microbial biocontrol solutions based on plant hormones (Shigenaga et al., 2017). Therefore, a detailed overview is required to better understand the multifaceted roles of phytohormones in regulating plant defenses and fitness (Vos et al., 2013; Guo et al., 2018). This will facilitate the optimized use of the phytohormone network for practical applications in plant cultivation and protection.

## 5 ACC deaminase mediated suppression of abiotic stress in plants

The enzyme 1-aminocyclopropane-1-carboxylate (ACC) deaminase is present in many PGPR, regulating plant growth by reducing the ethylene level produced in response to stress signals (Ali and Kim, 2018). Ethylene is a gaseous phytohormone that regulates plant growth at optimal concentrations; however, at higher concentrations, it affects various plant developmental processes, including root growth, nodulation, fruit ripening, flowering, and leaf senescence (Vanderstraeten et al., 2019). Prolonged exposure to various stresses can lead to increased accumulation of ethylene, which can significantly impact plant developmental processes (Dubois et al., 2018). IAA may elicit ACC synthesis, which severs as an intermediate precursor to ethylene biosynthesis and act as a key milestone in the regulation of ethylene production in plants.

**Figure 4:**
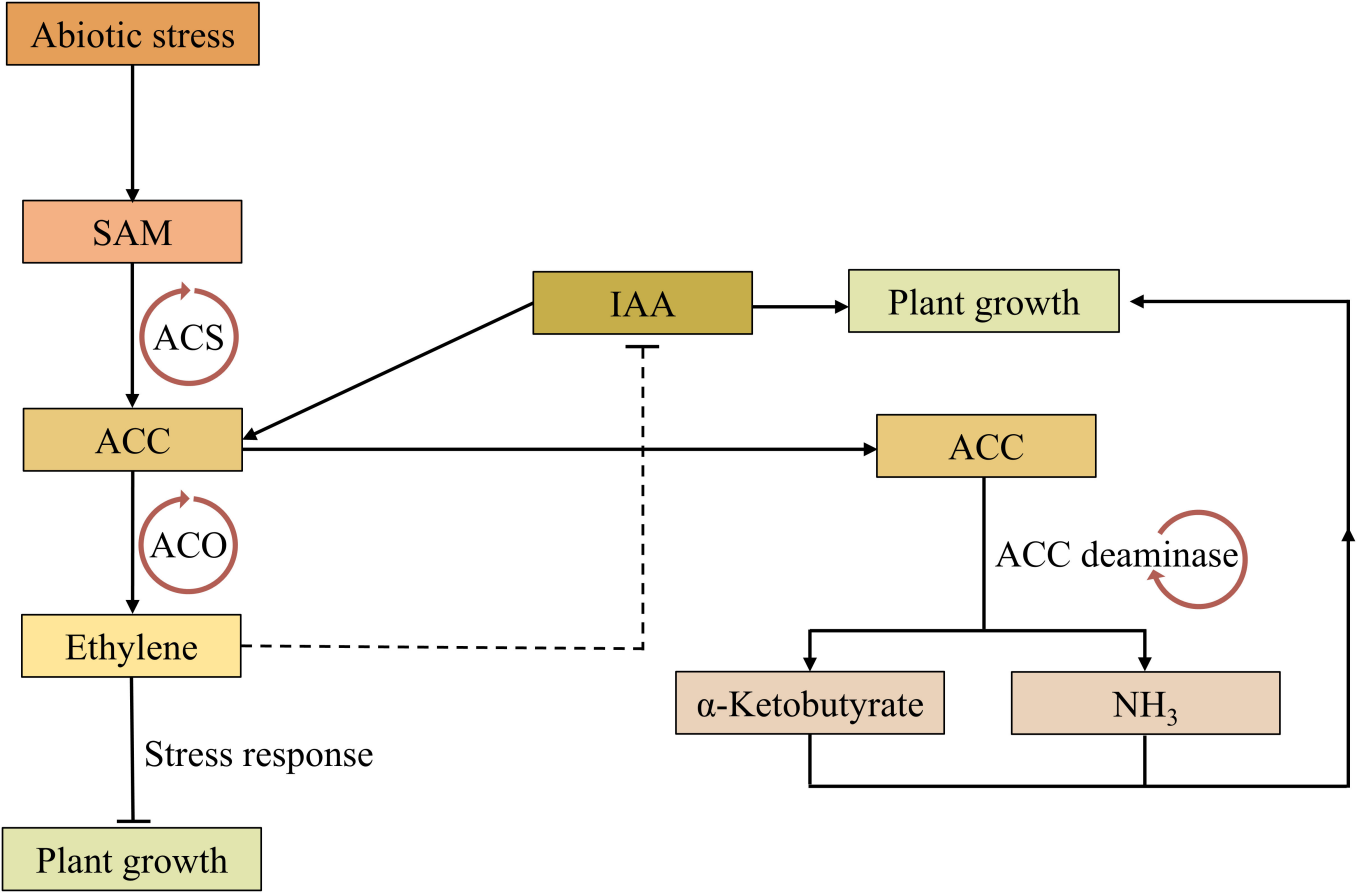
A schematic model proposing how ACC deaminase-producing bacteria promote plant growth by reducing ethylene concentration. The stress response in plants initiates with SAM conversion to ACC by the enzyme ACC synthase. ACC is subsequently converted to ethylene through ACC oxidase, which can hinder plant growth and restrict IAA biosynthesis. Meanwhile, ACC conversion in bacteria proceeding through ACC deaminase, resulting in the synthesis of α-ketubutyrate and ammonia, which in turn diminishes the synthesis of ethylene and promote plant growth. IAA biosynthesis is a complex and multi-enzymes/proteins process. Lines with an arrowhead represent a positive effect while those with flattened head represent inhibition. SAM, S-adenosyl-L-methionine; ACC, 1-aminocyclopropane-1-carboxylate; ACS, ACC synthase; ACO, ACC oxidase; IAA, indole-3-acetic acid.

When ACC is secreted by plant roots in the presence of high concentrations of ethylene, ACC deaminase-producing bacteria catalyze the breakdown of α-ketobutyrate and ammonia rather than ethylene, thereby reducing plant growth-inhibitory ethylene levels in developing or stressed plants (Ratnaningsih et al., 2023). Furthermore, IAA synthesis by these bacteria enhances root development and nutrient uptake, further supporting plant resilience against several abiotic stresses (**Figure 4**) (Etesami and Glick, 2024). Specifically, the role of IAA in promoting root and shoot growth significantly enhances plant adaptation to heavy metal stress (Shah et al., 2024). For instance, the availability of IAA has been shown to improve plant growth under heavy metal stress by increasing the phytoextraction of Pb, Zn, and Cd (Sytar et al., 2019). Similarly, the application of IAA can enhance plant growth in metal contaminated soil by mitigating the hazardous effects of heavy metals on plants (Sharif et al., 2022). Another study reported that the application of IAA-producing fungal endophyte *Penicillium roqueforti* greatly reduced the uptake of heavy metals by wheat plants (Ikram et al., 2018). Altogether, the synthesis of IAA by microbes plays significant role in enhancing plant growth and resilience, particularly in metal-contaminated soils.

Inoculating plants with ACC deaminase-producing bacteria provides protection against various stresses (Herpell et al., 2023; Roy Choudhury et al., 2023). Notably, *Trichoderma* strains containing ACC deaminase have been found to show phytopathogenic biocontrol and plant growth regulation activity in mangrove seedlings (Saravanakumar et al., 2018). Additionally, Increased ACC deaminase activity and IAA production was observed in the presence of *Serratia* K120 bacterium under heavy metal stress (Carlos et al., 2016). Similarly, it has been reported that *Paraburkholderia dioscoreae* Msb3, a novel bacterium designated strain, interacts with other symbionts, enhancing plant growth in tomato through ACC deaminase activity (Herpell et al., 2023). While the importance of ACC deaminase in plant growth promotion and abiotic stress resilience have been demonstrated, there is a lack of comprehensive studies on the plant root ACC exudation and microbial ACC deaminase activity under various abiotic stresses. In-depth investigation of the plant responses following the utilization of IAA- and ACC deaminase-producing bacteria would be rewarding, especially to reveal the best strategy for PGPR application in crop production, leading to more sustainable practices that reduce reliance on chemical fertilizers for better ecological safety.

## 6 Current challenges for optimizing plant-microbe interaction

Plants have been significantly affected due to the abrupt changes in climatic conditions, resulting in severe impacts on cellular homeostasis. These impacts eventually lead to stunted plant growth and development, highlighting the emerging need to shift from conventional breeding to more advanced and sustainable approaches. To address these challenges genetic engineering has emerged as a promising solution for developing microbial strains that are effective and possess extended lifespans to achieve crop yield under drought conditions. Recent developments in microbiology, molecular biology, and biotechnology have led to the discovery of novel genes linked to drought tolerance (Salvi et al., 2022). The integration of microbiotechnology concepts in agriculture should be leveraged to isolate microbial strains from the stress-affected soils, and further evaluation of these strains based on their stress tolerance may be useful in the process of bio-inoculation of crops cultivated in drylands (Kaushal and Wani, 2016).

Exploring diverse microbial communities poses a significant challenge in plant-microbe interaction research, as it is difficult to address important questions like the basic characteristics of a specific microbial community, the complex interplay among different community members, and how these members contribute to the survival of plants under such circumstances. It is now well-recognized that plants can regulate the recruitment of various root-associated microorganisms for required purposes (Bag et al., 2022). Moreover, the mechanisms of various signaling pathways in the rhizosphere that lead to the assembly and stability of intrinsic microbiome require investigation. Recent research breakthroughs in the area of plant-microbe interactions may have a significant impact on agricultural productivity. Research on these beneficial plant-microbe interactions in the rhizosphere will further elucidate how these microbes impact the nutritional composition of plant materials. However, it remains a challenging and demanding area of research to systematically exploit and utilize these beneficial microbial species living in the plant rhizosphere. Therefore, there is an urgent need to develop new and advanced scientific approaches to comprehend the complex interplay between plants and the microbiome under changing environmental conditions.

## 7 Recent innovations in plant-microbe interaction

The rhizosphere is an ever-changing environment, offering diverse and intriguing aspects to researchers. The advent of highly sophisticated molecular biology techniques has brought a new era for thoroughly investigating the complex plant-microbe crosstalk for green efficient production. The emergence and continuous updating of omics, gene-editing techniques, and high-throughput sequencing technology have opened up new ideas for studying the complex networks of plant-microbe interactions and plant resilience to different biotic and abiotic stresses (Kimotho and Maina, 2024). Genomics has proven to be an efficient tool in investigating and predicting plant-microbe interactions, as well as enhancing plant resilience to different stresses (Frantzeskakis et al., 2020). Multiple sequencing technologies, including prokaryotic 16S amplicon sequencing (Qin et al., 2024), fungal internal transcribed spacer (ITS) regions sequencing (Labouyrie et al., 2023), and metagenomics (Masuda et al., 2024) have been employed to analyze the extensive genetic variability present within the soil microbiome.

Next generation sequencing (NGS)-based transcriptomics is the most comprehensive and efficient approach to uncover the molecular background of plant-microbe interactions (Katara et al., 2024). It is mostly employed to evaluate plant performance under various stress conditions, revealing the physiological responses of plants to pathogens and elucidating the signaling mechanisms occurring in the rhizosphere (Roy et al., 2024). NGS has paved the way to investigate beneficial plant-microbe interactions and plant performance under abiotic stresses (Rehman et al., 2024). For instance, enhanced drought tolerance in wild soybean (Kim et al., 2024), the cold tolerance response in upland cotton (Wang et al., 2024), the impact of short-term flooding on the expression profile of orchard grass genes (Qiao et al., 2020), and altered gene expression level in Arabidopsis under cold stress (Zhang et al., 2024), have been revealed through NGS. Metagenome sequencing using oxford nanopore technologies (ONT) is currently the most effective strategy for pathogen detection (Yu et al., 2023). It is a robust and direct method of long-read sequencing without the need for an amplification step (Wang et al., 2021b). Due to its ability to directly detect pathogens except RNA viruses, it can be used without prior knowledge of pathogens (Javaran et al., 2023). The MinONTM technology has already been used for metagenome sequencing of bacteria, fungi, and viruses, affecting various crops (Jongman et al., 2020; Mechan Llontop et al., 2020). Overall, these modern molecular techniques have greatly improved agricultural productivity and sustainability with enhanced resilience to various ongoing climatic challenges.

## 8 Future perspectives

Plants, and specifically the rhizosphere are bustling with microorganisms. When plants encounter unfavorable conditions, they can recruit beneficial microbes to help mitigate these stresses by releasing a range of chemical signals, which is known as the ‘cry for help’ strategy (Bai et al., 2022). To understand the mechanistic background of ‘cry for help’ strategy of plants, it is important to investigate these molecular aspects of plant-microbe interactions in the rhizosphere (Zancarini et al., 2021). Identifying genes with specific functions is mandatory for understanding the signaling cascades that regulate plant growth and stress responses (Depuydt and Vandepoele, 2021). Understanding the interplay between plant functional genes and rhizosphere microbes will be essential for regulating plant growth and development processes, including root architecture, microbial abundance, phytohormones, secondary metabolites, nutrient acquisition, and plant immune responses (Liu et al., 2023). For instance, the MYB72 transcription factor in Arabidopsis, recognized for its prominent role in the induced systematic response (ISR) mediated by beneficial microbes, represents a promising area for further investigation (Vlot et al., 2021). Additionally, the roles of members of the plant multidrug and toxic compound extrusion (MATE) family in transporting phytohormones and secondary metabolites require further exploration (Wang et al., 2022). Moreover, the co-expression of the Arabidopsis gene *AVP1*, the rice gene *OsSIZ1*, and the cyanobacterium flavodoxin gene *FId* revealed their important contributions to plant growth and resilience to multiple environmental adversities through delicately fine-tuned their genetic and epigenetic regulation (Zhao et al., 2024).

Innovations in plant-microbe interactions at the molecular level offer a new direction for genetic breeding (Nerva et al., 2022). Exploring this relationship will help us develop crop varieties with enhanced adaptability to various stresses. Future research using cutting-edge technologies such as multi-omics, NGS, and imaging techniques as discussed in section 7, will enhance our understanding of the microbes that mutually interact with host genes is expected to provide new germplasm resources. Furthermore, the molecular and mechanistic background of microbial ACC deaminase activity in response to root-exuded ACC under various abiotic stresses is still at a preliminary stage. In-depth investigation of plant responses to IAA- and ACC deaminase-producing bacteria would be rewarding, especially in revealing the best strategies for PGPR application in crop production, leading to more sustainable practices that reduce reliance on chemical fertilizers for better ecological safety.

## 9 Conclusions

Climate change, with increased adversity, is a concurrent global concern that leads to implications for worldwide food security, negatively impacting both plant and microbial growth. Identifying innovative approaches to meet the growing demand for food in a changing climate is challenging, particularly as plant stresses and diseases greatly affect crop production and sustainability. Improving plant adaptations to these stressors while increasing agricultural production is one of the potential demands. In this context, the current review explores comprehensive insights into how plants and their microbiomes enhance resilience to stresses like drought, salinity, and temperature variations. We highlight key microbial strategies that mediate plant tolerance, including ROS scavenging, antioxidant regulation, and IAA synthesis. Additionally, we emphasize the role of ACC deaminase-producing bacteria in regulating ethylene levels, suggesting that future research should focus on the interaction between plant root ACC exudation and microbial ACC deaminase activity for effective ethylene mitigation during stress. Recent advancements in molecular techniques, such as next-generation sequencing and multi-omics approaches, are discussed to optimize these interactions, along with the chemotactic movement of microbes driven by hormonal crosstalk and root exudation.
